# Deposition of Multilayer Nanostructured Coating Cr/(Cr/a-C)ml on Alloy Steels

**DOI:** 10.3390/ma18214923

**Published:** 2025-10-28

**Authors:** Boyan Dochev, Yavor Sofronov, Valentin Mishev, Antonio Nikolov, Krum Petrov, Milko Angelov, Milko Yordanov, Georgi Todorov, Krassimir Marchev

**Affiliations:** 1 Department of Mechanics, Faculty of Mechanical Engineering, Technical University of Sofia—Branch Plovdiv, 4000 Plovdiv, Bulgaria; 2 Department of Theory of Mechanisms and Machines, Faculty of Industrial Technology, Technical University of Sofia, 1756 Sofia, Bulgaria; 3 Department of Material Science and Technology of Materials, Faculty of Industrial Technology, Technical University of Sofia, 1756 Sofia, Bulgaria; v_mishev@tu-sofia.bg (V.M.); anikolov@tu-sofia.bg (A.N.); kpetrov@tu-sofia.bg (K.P.); 4 Faculty of Industrial Technology, Technical University of Sofia, 1756 Sofia, Bulgaria; milko.angelov@tu-sofia.bg (M.A.); k.marchev@northeastern.edu (K.M.); 5 Department of Mechanical Engineering, Manufacturing Engineering and Thermal Engineering, Faculty of Engineering and Pedagogy—Branch Sliven, Technical University of Sofia, 8800 Sliven, Bulgaria; m_yordanov@tu-sofia.bg; 6 Center of Excellence “Mechatronics and Clean Technology”—Campus Studentski Grad, Technical University of Sofia, 1756 Sofia, Bulgaria; gdt@tu-sofia.bg; 7 Department of Manufacturing Technology and Systems, Faculty of Industrial Technology, Technical University of Sofia, 1756 Sofia, Bulgaria; 8 College of Professional Studies, Northeastern University, Boston, MA 02115, USA

**Keywords:** alloy steels, nanostructured coating, characteristics of Cr/(Cr/a-C)ml coating, nanolayered coating

## Abstract

A chromium/amorphous carbon (Cr/(Cr/a-C)ml) nanostructured multilayer coating with a chromium sublayer was deposited on 42CrMo4 (1.7225,BDS EN ISO 683-2:2018), 100Cr6 (1.3505, BDS EN ISO 683-17:2024), and HS18-0-1 (1.3355, BDS EN ISO 4957:2018) alloy steels, selected for their use in contact-loaded components subjected to cyclic fatigue and intense wear. The coating was sputter deposited by MF pulsed magnetron sputtering under consistent process parameters. The resulting coating, approximately 1.8 μm thick, can significantly enhance the service life of these components. Adhesion was evaluated via the Daimler–Benz test, while coating homogeneity was confirmed through energy-dispersive spectroscopy, revealing a consistent chemical composition across sample surfaces. Raman spectroscopy indicated a high sp^3^/sp^2^ ratio, confirming a dominant diamond-like carbon structure. Nanoindentation measurements verified the coating’s hardness, aligning with the observed structural properties. These results validate the process parameters for depositing a Cr/(Cr/a-C)ml coating on these alloy steels, achieving this study’s objectives.

## 1. Introduction

During their operation, mechanical components and tools experience performance degradation due to wear, leading to undesirable operational consequences. To enhance properties such as wear resistance, thermal stability, and corrosion resistance, various coatings are deposited, including single-layer coatings (e.g., oxides, carbides, and nitrides of transition metals such as Al_2_O_3_, TiC, TiN, ZrN, and CrN) and advanced multilayer, nanocomposite, or nanolaminate structures [1,2,3,4,5,6,7,8,9,10,11,12,13,14,15,16].

Recent research has focused extensively on chromium/amorphous carbon (Cr/a-C) nanostructured multilayer coatings. They represent an advanced class of thin-film materials engineered at the nanoscale, which consist of alternating nanolayers of metallic chromium (Cr) and amorphous carbon (a-C) [1]. They are a type of diamond-like carbon (DLC) coating due to their tunable properties, governed by the sp^3^/sp^2^ phase ratio [2,3]. The sp^2^ phase contributes to low friction coefficients and electrical conductivity, while the sp^3^ phase enhances chemical inertness, hardness, and wear resistance, making DLC coatings highly promising for wear-resistant applications in mechanical engineering [2,17,18,19,20,21,22,23]. Additionally, DLC coatings are biocompatible, broadening their utility in medical applications [24,25,26]. Incorporating carbide-forming elements into DLC coatings can further enhance hardness and wear resistance by forming a nanolaminate structure [1,4,5]. However, high internal stress in DLC coatings may lead to delamination from substrates, posing a challenge.

**Figure 1:**
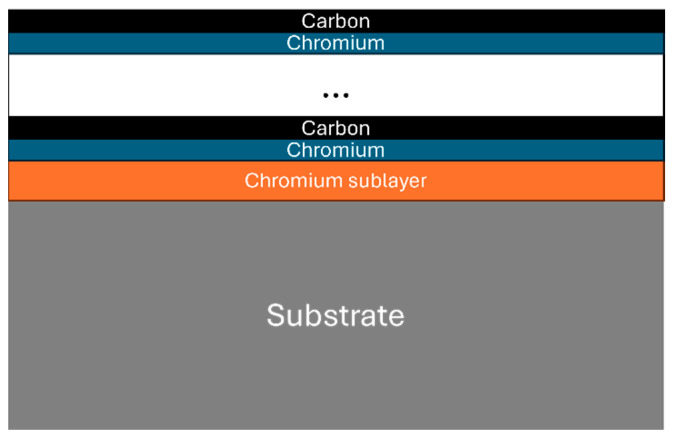
Chromium, chromium–carbon multilayer coating.

Common deposition techniques for hard coatings include physical vapor deposition (PVD) and chemical vapor deposition (CVD) [27,28,29,30,31,32,33,34]. Post-deposition, the technological and operational properties of coatings must be rigorously evaluated [35,36,37,38,39,40,41,42]. PVD is advantageous for depositing coatings with excellent adhesion and controlled morphology at relatively low temperatures (150–250 °C), preserving the structural integrity of the steel substrates. PVD enables the deposition of single-layer, multilayer, and complex coating systems; accommodates intricate geometries; and operates under moderate vacuum conditions. In contrast, CVD requires higher temperatures, potentially generating hazardous by-products and complicating selective coating applications. This study aims to validate specific process parameters for depositing a nanostructured multilayer Cr/(Cr/a-C)ml coating, as shown in Figure 1, by characterizing their resulting properties. Different alloy steel materials are used to evaluate the influence of substrate-dependent parameters such as growth layer thickness and coating adhesion performance on the substrate.

Analytical methods like Calotest layer thickness, SEM imaging, Daimler–Benz adhesion testing, EDX, nanoindentation, and Raman spectroscopy were used.

## 2. Materials and Methods

Three alloy steels—42CrMo4 (1.7225, EN 10083-3:2006) BDS EN ISO 683-2:2018 [43], 100Cr6 (1.3505, EN 683-17:1999) BDS EN ISO 683-17:2024 [44], and HS18-0-1 (1.3355, EN ISO 4957:2000) BDS EN ISO 4957:2018 [45]—were selected for their application in high-stress components subjected to cyclic fatigue and wear. These steels underwent quenching and tempering (QT) heat treatment to optimize their mechanical properties. 42CrMo4 is widely used in automotive and aerospace industries for components such as gears, axles, crankshafts, and landing gear, owing to its high strength and fatigue resistance. 100Cr6 is employed in rolling bearings, gears, and heavy machinery components due to its exceptional hardness, wear resistance, and dimensional stability. HS18-0-1, capable of retaining high hardness at temperatures up to 700 °C, is ideal for tools like drills, broaches, and punches.

Nine samples (3 from every steel) were prepared with ten grades of silicon carbide grinding papers starting with grade 220 (coarse) and finishing with grade 2000 (fine). Polishing was performed with a 1 µm diamond polishing agent (Struers GmbH, Willich, Germany) for 5 to 7 min up to mirror polishing. Specimens were initially subjected to ultrasonic cleaning in deionized water for 10 min, followed by treatment with Deconex solvent in an ultrasonic bath at 60 °C for 10 min. A subsequent rinse in deionized water was performed for 10 min to remove residual solvent contaminants. The samples were then dried with a controlled hot-air stream and further subjected to vacuum drying at 100 °C for 1 h to ensure complete removal of moisture.

**Figure 2:**
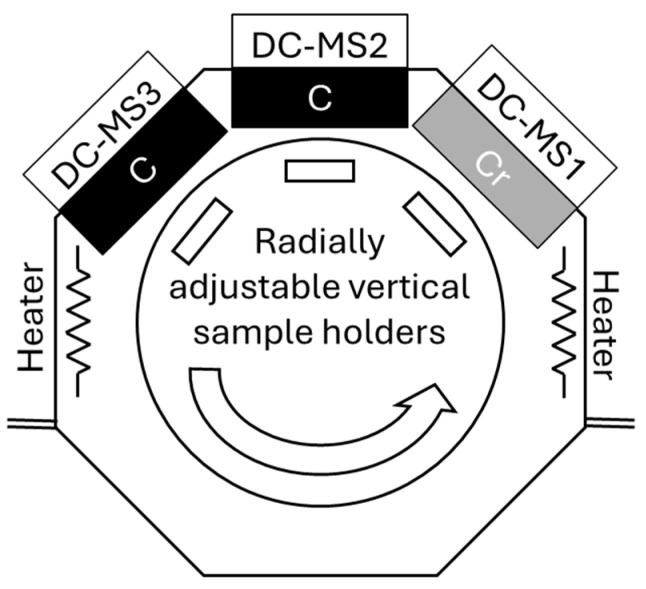
Developed proprietary magnetron sputtering system.

The Cr/(Cr/a-C)ml coating was deposited using a proprietary magnetron sputtering system developed at the Center of Excellence “Mechatronics and Clean Technologies” at the Technical University of Sofia. The system features an octagonal chamber with three unbalanced magnetrons equipped with rectangular targets (360 mm × 102 mm × 9 mm): two of high-purity carbon (99.99%) and one of chromium (99.8%). Samples were mounted on three holders on a single-axis rotating table, as shown in Figure 2. The deposition process was optimized through iterative trials.

The samples were adjusted at a 60 mm distance from the targets. Cr, as the sublayer and as the nanolamination material, was selected because it increases the corrosion resistance of the coating by forming a dense, passive chromium oxide layer on the surface, which acts as a physical barrier against oxygen and water. Carbon as a nanolamination material is used to improve coating hardness and respective wear resistance of the coated components. It also contributes to decreasing the friction coefficient.

The magnetron sputtering process involved three stages: (1) substrate cleaning by glow discharge at 3 Pa and 235–240 °C in an Ar–H_2_ mixture at 1000 V MF pulsed bias for 15 min; (2) substrate cleaning with chromium ions at 3 × 10^−1^ Pa, 1000 W on the chromium target and −1000 V bias at 225–230 °C for 15 min; and (3) deposition of the chromium adhesion layer at 2.6 × 10^−1^ Pa and 1800 W for 8 min, followed by the Cr/(Cr/a-C)ml coating. The latter involved powering carbon targets at 250 W for 1 min, increasing to 750 W, while reducing chromium target power from 1800 W to 700 W over 10 min and lowering substrate temperature to 160 °C. The nanostructured multilayer coating was deposited over 180 min, yielding a smooth, uniform, and dense coating without visible defects.

Adhesion was assessed via a DIN 4856:2018-02 [46] standardized Rockwell indentation test for the evaluation of the adhesion of coatings, known as the Daimler–Benz test. It utilizes a Rockwell C indenter (120° diamond cone, 200 nm tip radius, 1471 N load) on a VEB WPM Leipzig device (Leipzig, Germany), with the crater measured using a “Best Scope BS-6022TRF” (Beijing Bestscope Technology Co., Ltd., Beijing, China) microscope and Capture 2.1 software. The degree of damages is classified into HF1–HF6 adhesion grades, based on crack patterns and delamination. Chemical composition was analyzed via scanning electron microscopy (SEM, EVO MA 10, Carl Zeiss, Oberkochen, Germany) with energy-dispersive X-ray spectroscopy (EDS, Bruker, Billerica, MA, USA). Hardness was measured by nanoindentation using a Shimadzu DUH-211S tester (Kyoto, Japan) with a Berkovich indenter in Loading–Unloading mode (5 mN force, 0.4877 mN/s loading speed, 1 s hold time, 25 °C). Raman spectroscopy was performed using a LabRAM HR Visible spectrometer (Horiba Scientific, Palaiseau, France) with a 633 nm He–Ne laser (0.57 mW, ×100 objective) to assess carbon bonding.

## 3. Results

The characteristics of the deposited Cr/(Cr/a-C)ml coating were systematically evaluated using a suite of analytical techniques to validate the efficacy of the selected PVD process parameters. These evaluations encompassed thickness measurements, a surface morphology assessment, adhesion testing, a compositional analysis, hardness determination, and structural characterization via Raman spectroscopy. The results are presented below, highlighting consistency across the three substrate materials where applicable.

**Figure 3:**
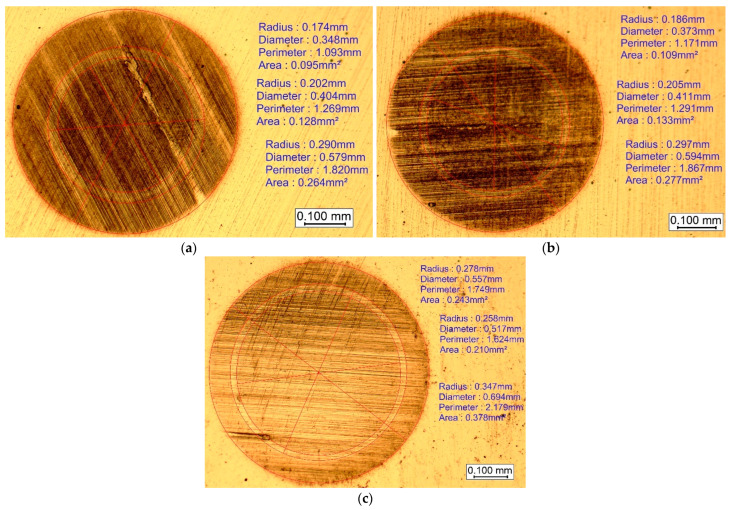
Measurements to determine the thickness of the individual layers after Calotest: (**a**) 42CrMo4 steel, (**b**) 100Cr6 steel, and (**c**) HS18-0-1 steel.

Coating thickness was measured using the Calotest method, according to the ISO 26423:2009 [47], with a KaloMAX II calotester (BAQ GmbH, Bremen, Germany) at 200 min^−1^ for 20 s using a 1 µm diamond abrasive suspension (BAQ GmbH, Bremen, Germany) and a nitrided 100Cr6 steel sphere with a diameter of 30 mm. These measurements were derived from an optical analysis of the wear craters (Figure 3a–c), demonstrating uniform deposition across the samples despite minor variations attributable to substrate-specific interactions during growth. The range of layer thicknesses measured by the Calotest method was approximately from 0.3 to 50 µm, and the measurement accuracy was affected by surface roughness, but measurement accuracy was from 1 to 5%. The analysis was performed at a minimum of three different regions, and the average thickness is represented in Table 1.

**Figure 4:**
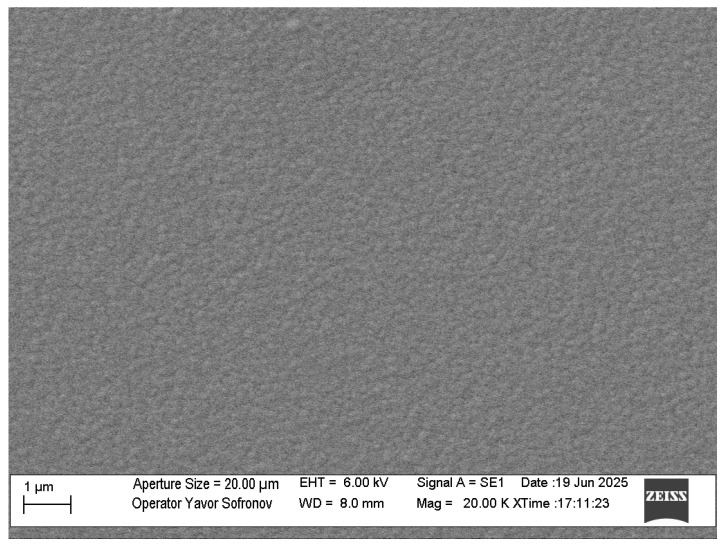
Coating surface of 42CrMo4 sample observed by SEM imaging.

Surface morphology was examined via SEM, revealing a compact, smooth coating with low surface roughness across all substrates. This uniformity minimizes friction in tribological systems and enhances overall performance. Representative imaging of the 42CrMo4 substrate (Figure 4) showed a dense structure with negligible defects, such as isolated nanodroplets, which are common in PVD processes but do not compromise integrity here. The absence of material-dependent variations in surface quality underscores the robustness of the deposition parameters.

**Figure 5:**
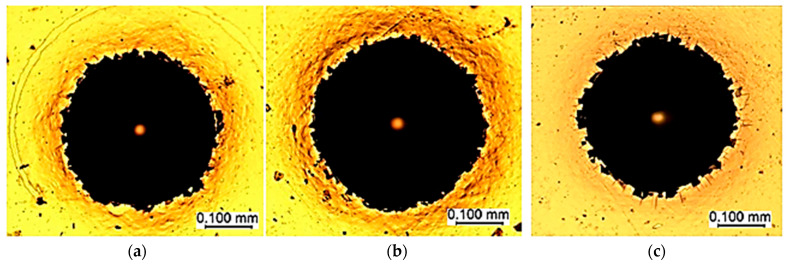
Evaluation of coating adhesion using the Daimler–Benz adhesion test: (**a**) 42CrMo4 steel, (**b**) 100Cr6 steel, and (**c**) HS18-0-1 steel.

Adhesion quality, a critical factor for coating durability, was assessed using the Daimler–Benz Rockwell C indentation test. For the 100Cr6 and HS18-0-1 substrates, the coating exhibited minor radial cracking without significant delamination, corresponding to adhesion classes HF2–HF3 (Figure 5b,c)—levels considered acceptable for industrial applications. In contrast, the 42CrMo4 substrate displayed diametrically opposed cracks extending from the indentation perimeter (Figure 5a), indicative of slightly reduced adhesion and classification as HF4. These observations align with known challenges in achieving optimal DLC adhesion on certain steel substrates, potentially influenced by substrate hardness and surface energy.

**Figure 6:**
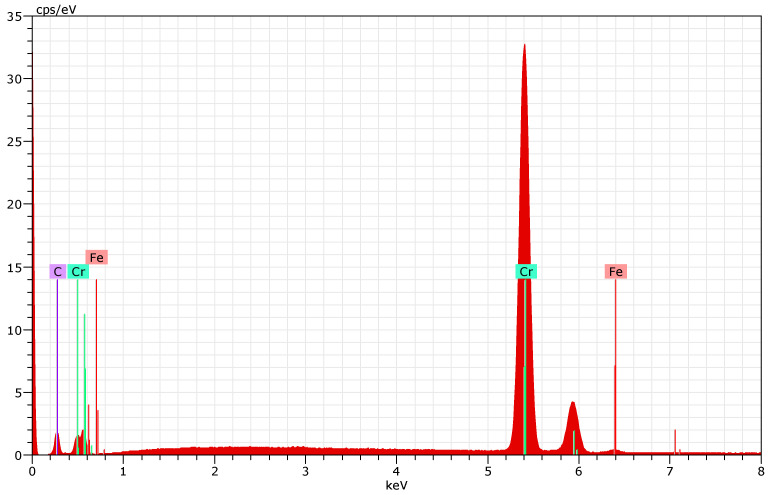
Determination of the coating composition by EDX on 100Cr6 steel.

Chemical composition and compositional homogeneity were confirmed through EDS analysis at a few surface locations for all the samples. The results for elemental distributions of 100Cr6 steel with a (Cr/a-C)ml coating at three points are represented in Table 2. Spectra from distinct regions on representative samples showed consistent elemental distributions, with chromium at approximately 69.73 at% and carbon at 29.61 at% (Figure 6).

**Figure 7:**
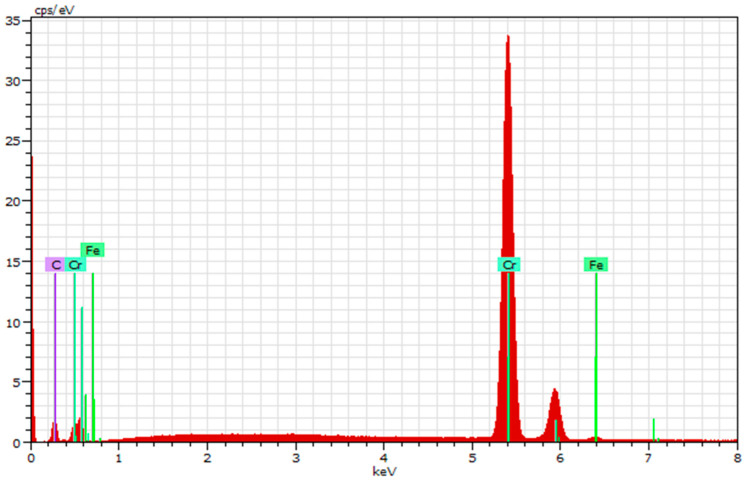
Determination of the coating composition by EDX on 42CrMo4 steel.

**Figure 8:**
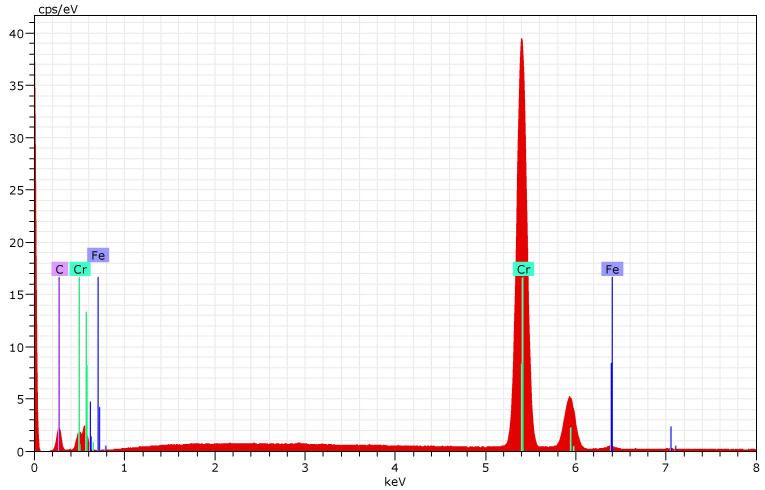
Determination of the coating composition by EDX on HS18-0-1 steel.

The same analyses were performed for the 42CrMo4 (Figure 7) and HS18-0-1 (Figure 8) steel samples, and the results exhibit similar values for their elemental composition because it is the coating parameter. Minor deviations (e.g., trace oxygen or other elements) were negligible and likely artifacts from sample environmental exposure. This uniformity of the coating ensures isotropic properties, essential for reliable performance under operational stresses.

**Figure 9:**
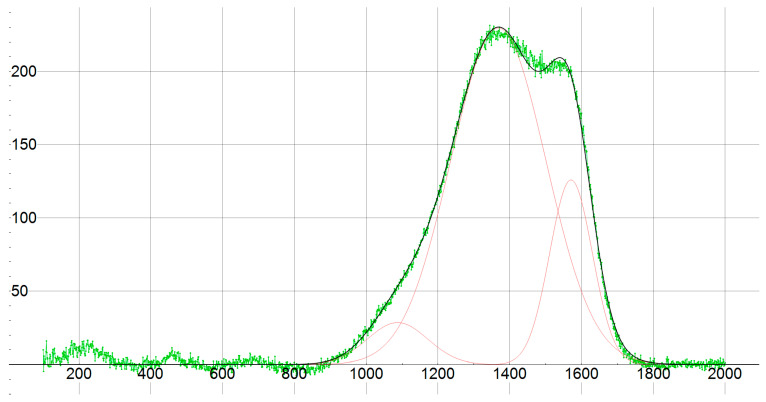
Raman spectrum of material 42CrMo4.

Structural analysis via Raman spectroscopy elucidated the carbon hybridization state, a key determinant of DLC properties. Spectra from all samples displayed characteristic broad D (1367–1368 cm^−1^) and G (1570–1571 cm^−1^) bands, indicative of amorphous carbon with mixed sp^2^ and sp^3^ bonding (Figure 9 for 42CrMo4 representative). Deconvolution yielded an sp^3^/sp^2^ intensity ratio of 1.82 (Table 3), signifying a predominance of diamond-like (sp^3^) phases that contribute to elevated hardness and wear resistance. This high ratio, consistent across substrates, validates the process’s ability to favor sp^3^ formation under the deposition conditions. Such a high ratio is a significant scientific contribution of magnetron sputtering [2] and represents the excellence of the design of the developed proprietary system.

Mechanical properties were evaluated via nanoindentation, providing insights into hardness and elastic behavior. Measurements across all substrates yielded a consistent indentation hardness (Hit) of 33.3 ± 3.6 GPa, exceeding the threshold for hard coating (>20 GPa) and confirming suitability for friction-wear environments. Detailed data from the HS18-0-1 substrate (Table 4) illustrates variability within individual indents (e.g., Hit ranging from 28.6 GPa to 38.9 GPa), but the average aligns closely with the overall results. Parameters such as maximum penetration depth (hmax ≈ 0.104 μm), plastic depth (hp ≈ 0.057 μm), and elastic modulus (Eit ≈ 476 GPa) further characterize the coating’s resistance to deformation.

Overall, the proprietary magnetron sputtering system and parameters produced a coating with reproducible thickness, composition, and properties, demonstrating process stability and the potential for scalability in engineering applications.

The challenge for the scientific team is to develop process parameters to achieve a coating of a different composition, optimize the technological parameters, and evaluate their characteristics as well as their practical implementation.

## 4. Discussion

The successful deposition of a Cr/(Cr/a-C)ml nanostructured multilayer coating via magnetron sputtering on three distinct alloy steel substrates highlights the versatility and control afforded by the optimized process parameters. The coating exhibited comparable total thicknesses of ~1.8 μm across substrates with varying chemical compositions, affirming the method’s independence from minor substrate differences in terms of overall growth rate. However, subtle variations in the chromium adhesion sublayer thickness—thicker on HS18-0-1 (0.360 μm) compared to 100Cr6 (0.223 μm) and 42CrMo4 (0.314 μm)—suggest potential influences from substrate chromium content or surface reactivity (Table 1). HS18-0-1, with the highest inherent chromium concentration, may facilitate enhanced nucleation or diffusion during the initial deposition stage, leading to a thicker adhesion layer. Conversely, the correspondingly thinner (Cr/a-C)ml layer on HS18-0-1 could result from mass balance effects or altered sputtering dynamics. While a definitive causal link between substrate composition and layer thickness remains elusive, these observations warrant targeted future studies, such as in situ monitoring of growth kinetics or comparative depositions on chromium-modified surfaces.

Adhesion performance, as gauged by the Daimler–Benz test, revealed substrate-dependent behavior that correlates strongly with macrohardness. The superior HF2–HF3 classification for 100Cr6 (61 HRC) and HS18-0-1 (63 HRC) contrasts with the HF4 rating for 42CrMo4 (57 HRC), where extended cracks indicate incipient delamination (Figure 5). This disparity underscores the role of substrate mechanical support in mitigating stress-induced failures at the coating interface. Softer substrates like 42CrMo4 may deform more readily under indentation loads, exacerbating tensile stress and crack propagation. These findings resonate with the literature on DLC coatings, where internal compressive stresses—arising from sp^3^ bonding—can promote delamination unless counterbalanced by robust interfacial bonding [17,18,19,20,21,22,23]. The chromium adhesion layer likely serves as a stress-relief buffer, but its efficacy appears modulated by substrate hardness, suggesting opportunities for enhancement through graded interfaces or alternative interlayers.

Compositional uniformity, evidenced by EDS (Figure 6, Figure 7 and Figure 8), ensures consistent mechanical properties, with the ~64 at% Cr and ~32 at% C ratios promoting carbide formation that bolsters hardness. The measured Hit of 33.3 ± 3.6 GPa positions this coating among high-performance DLC variants, attributable to the high sp^3^/sp^2^ ratio (1.82) confirmed by Raman spectroscopy. This ratio implies a diamond-like matrix with embedded chromium carbide nanocrystallites, enhancing load-bearing capacity while maintaining low friction—key for cyclic fatigue applications. Variations in nanoindentation data (Table 3) reflect localized heterogeneities, such as minor defects or phase distributions, but the narrow standard deviation supports overall homogeneity.

In comparison to reported PVD-DLC coatings [27,28,29,30,31,32,33,34,35,36], the achieved properties demonstrate competitive advantages, including low-temperature deposition that preserves substrate microstructure and biocompatibility potential [24,25,26]. Challenges like adhesion on softer steels align with known limitations, potentially addressable via process refinements (e.g., bias voltage adjustments) or substrate pretreatments. The SEM-observed low roughness and defect density further indicate tribological superiority, reducing wear initiation sites. Collectively, these results validate the proprietary system’s efficacy and highlight pathways for tailoring coatings to specific substrate demands, contributing to advancements in wear-resistant technologies for mechanical engineering.

## 5. Conclusions

The application of hard, wear-resistant, and corrosion-resistant coatings represents a cornerstone strategy for improving the durability and functionality of alloy steel components in demanding engineering contexts. In this study, the multilayer nanostructured Cr/(Cr/a-C)ml coating, deposited via an optimized PVD process, exhibited exemplary characteristics: a uniform total thickness of approximately 1.8 μm, homogeneous composition (~64 at% Cr, ~32 at% C), and superior hardness (Hit = 33.3 ± 3.6 GPa). These attributes, coupled with a high sp^3^/sp^2^ ratio of 1.82, affirm the coating’s diamond-like carbon dominance, which imparts enhanced mechanical integrity and tribological performance suitable for contact-loaded parts subjected to cyclic fatigue and abrasion.

The process parameters, refined through iterative development on a proprietary magnetron sputtering system, demonstrated reproducibility across diverse substrates (42CrMo4, 100Cr6, HS18-0-1), with minor variations in adhesion layer thickness and adhesion quality attributable to substrate hardness and composition. Notably, stronger adhesion (HF2–HF3) on harder steels underscores the importance of mechanical compatibility at the interface, while uniform surface morphology and minimal defects ensure operational reliability.

These findings not only validate the selected deposition regime but also underscore the potential of Cr/(Cr/a-C)ml coatings to extend service life in industries such as automotive, aerospace, and tooling.

## Figures and Tables

**Table 1:** Calculated thicknesses of the sublayer, (Cr/a-C)ml coating, and total and average thickness of the PVD coating.

Substrate Material	Sublayer	(Cr/a-C)ml Coating	PVD Coating Total Thickness	PVD Coating Average Thickness
42CrMo4	0.264 µm 0.351 µm 0.328 µm	1.571 µm 1.434 µm 1.539 µm	1.835 µm 1.784 µm 1.868 µm	1.829 ± 0.03 µm
100Cr6	0.143 µm 0.248 µm 0.278 µm	1.611 µm 1.533 µm 1.461 µm	1.754 µm 1.781 µm 1.738 µm	1.758 ± 0.016 µm
HS18-0-1	0.299 µm 0.358 µm 0.422 µm	1.462 µm 1.428 µm 1.301 µm	1.761 µm 1.786 µm 1.722 µm	1.756 ± 0.023 µm

**Table 2:** Calculated elemental distributions of 100Cr6 steel with (Cr/a-C)ml coating.

Element	Atomic %	Atomic %	Atomic %	Atomic % Average
Cr	69.33	69.73	69.52	69.53
C	29.96	29.61	29.80	29.79
Fe	0.71	0.65	0.68	0.68

**Table 3:** Raman spectrum results of material 42CrMo4.

Center	Intensity
1368	229.597
1571	125.859
Sp^3^/Sp^2^ (Int) = 1.82	

**Table 4:** Measured nanohardness on sample from HS18-0-1 material.

SEQ	Fmax [mN]	hmax [µm]	hp [µm]	hr [µm]	HMT115 [N/mm^2^]	HMs [N/mm^2^]	Hit [N/mm^2^]	Eit [N/mm^2^]	Cit [%]	nit [%]	HV+	Rer [%]
1	5.08	0.1033	0.0633	0.0804	1803.787	11,561.121	28,616.229	685,300	2.122	33.21	2644.139	38.803
2	5.08	0.1093	0.061	0.0765	1610.883	12,162.525	29,555.637	422,400	3.491	44.752	2730.941	42.925
3	5.09	0.1093	0.0523	0.0674	19,126.373	15,543.851	37,142.172	483,900	1.314	50.055	3431.937	46.855
4	5.08	0.1058	0.0541	0.0714	17,188.176	13,111.882	33,171.613	437,100	2.306	47.967	3065.057	48.104
5	5.08	0.1078	0.0511	0.0656	18,544.305	16,019.188	38,055.906	434,000	1.088	51.413	3516.366	48.822
6	5.08	0.097	0.0501	0.0661	20,427.801	12,314.193	38,885.379	558,600	0.951	47.408	3593.009	47.002
7	5.08	0.1096	0.0621	0.0731	16,012.796	15,338.083	31,361.984	378,000	1.997	44.161	2897.847	41.583
8	5.09	0.1074	0.0584	0.0764	16,691.564	14,679.676	29,989.211	458,800	1.549	45.573	2771.003	44.107
9	5.09	0.1043	0.0553	0.0707	17,888.674	10,897.512	33,946.418	443,800	1.929	46.987	3136.649	46.446
10	5.09	0.1056	0.0594	0.0736	17,525.846	12,629.287	31,898.174	458,400	2.343	43.574	2947.391	41.902
Average	5.08	0.1044	0.0567	0.0721	17,700.49	13,065.732	33,262.273	476,000	1.863	45.51	3073.434	44.755
Std Dev	0.001	0.004	0.005	0.005	1387.461	1761.189	3676.32	86,974.93	0.746	4.997	339.692	3.101
CV	0.024	3.804	8.405	6.739	7.802	13.49	11.053	18.271	9.128	10.981	11.053	6.93

## Data Availability

The original contributions presented in this study are included in the article. Further inquiries can be directed to the corresponding authors.

## Abbreviations

PVD	Physical vapor deposition
CVD	Chemical vapor deposition
(Cr/a-C)ml	Chromium/amorphous carbon multilayer
DLC	Diamond-like carbon
EDS	Energy dispersive spectroscopy
SEM	Scanning electron microscope
MF	Medium frequency
